# Alendronate Use and Risk of Type 2 Diabetes: A Nationwide Danish Nested Case-Control Study

**DOI:** 10.3389/fendo.2021.771426

**Published:** 2021-11-19

**Authors:** Rikke Viggers, Zheer Al-Mashhadi, Jakob Starup-Linde, Peter Vestergaard

**Affiliations:** ^1^ Steno Diabetes Center North Jutland, Department of Endocrinology, Aalborg University Hospital, Aalborg, Denmark; ^2^ Department of Clinical Medicine, Aalborg University, Aalborg, Denmark; ^3^ Steno Diabetes Center Aarhus, Aarhus University Hospital, Aarhus, Denmark; ^4^ Department of Clinical Medicine, Aarhus University, Aarhus, Denmark; ^5^ Department of Endocrinology and Internal Medicine, Aarhus University Hospital, Aarhus, Denmark

**Keywords:** diabetes, type 2 diabetes, bone, alendronate, bisphosphonate

## Abstract

**Objective:**

A link has been proposed between glucose homeostasis and bone metabolism. Bisphosphonates are first-line treatment of osteoporosis, and we aimed to investigate whether the risk of developing type 2 diabetes was associated with prior use of alendronate.

**Research Design and Methods:**

We conducted a population-based nested case-control study through access to all discharge diagnoses (ICD-10 system) from the National Danish Patient Registry along with all redeemed drug prescriptions (ATC classification system) from the Health Service Prescription Registry. All cases with a diagnosis of type 2 diabetes between 2008 and 2018 were matched on sex and age with 3 randomly selected controls by incidence-density sampling. Exposure was defined as ever use of alendronate and further grouped as effective and compliant use. ORs were calculated by conditional logistic regression analysis with adjustment for several confounders and test for trend for dose-response relationship.

**Results:**

We included 163,588 patients with type 2 diabetes and 490,764 matched control subjects with a mean age of 67 years and 55% male subjects. The odds of developing type 2 diabetes were lower among ever users of alendronate (multiple adjusted OR: 0.64 [95% CI 0.62-0.66]). A test for trend suggested a dose-response relationship between longer effective use of alendronate and lower risk of type 2 diabetes.

**Conclusion:**

These results suggest a possible protective effect of alendronate in a dose-dependent manner against development of type 2 diabetes.

## Introduction

Type 2 diabetes and osteoporosis are emerging global health problems with increased morbidity and mortality and continuous demand for disease prevention and management (1, 2). Bisphosphonates, e.g. alendronate, are first-line treatment of osteoporosis; the treatment sufficiently suppresses bone resorption with few adverse effects (3).

Antiresorptive therapies, e.g. alendronate, were initially hypothesized to decrease insulin sensitivity by decreasing osteocalcin levels (4). Contrarily, current research is pointing towards a possible protective effect of alendronate on the risk of developing type 2 diabetes as well as reducing insulin consumption (5–9). In this population-based study we hypothesized that alendronate use was not associated with the development of incident type 2 diabetes and examined a potential dose-dependent relationship.

## Research Design and Methods

The STROBE statement guideline for reports of case-control studies was followed (a STROBE checklist can be found in **Supplemental Table S1**) (10).

### Study Design and Setting

We conducted a population-based nested case-control study using information from Danish nationwide registries. Case subjects were people with type 2 diabetes, and controls were subjects without diabetes mellitus. For each case subject, 3 age- and gender-matched control subjects were randomly selected from the general population by incidence-density sampling (11). Exposure was use of alendronate prior to index date. As the time of data collection was set between January 1^st^ 1998 and December 31^st^ 2018. We estimated outcome (type 2 diabetes diagnosis) from January 1^st^ 2008 until December 31^st^ 2018 and exposure (alendronate use) from January 1^st^ 1998 until end of exposure or index date.

### Data Sources

Data were available and anonymized by Statistics Denmark (*Danmarks Statistik*, project identifier no. 703382) and were obtained through National Danish registers. All Danish citizens are assigned a 10-digit personal identification number (PIN) which ensures a complete medical history of all contacts to the Danish health care system and drug prescriptions for each individual (12–14). The unique PIN was anonymized and linked to all registries used in this study. All Danish citizens have equal access to full health care provided by the Danish National Health Service, which includes free access to hospitals and partial compensation of drug expenses. All authorized Danish research organizations can apply for access to the registries. An ethics committee approval is not required for epidemiological studies in Denmark, as we had no access to personally identifiable information. However, the registries are subject to control by the Danish Data Protection Agency.

Data on diagnoses were obtained from the Danish National Patient Registry (14). The registry covers all contacts to the hospitals on both in- and outpatient basis. The data include all relevant physician-assigned discharge diagnoses on individual level, coded according to the International Classification of Diseases, Tenth Revision (ICD-10).

Information on drug prescriptions were coded according to the Anatomical Therapeutical Chemical (ATC) classification and recorded from 1996 by the Danish National Health Service Prescription Registry (13, 15). To ensure adequate registration, we collected data from 1998.

Data on sex and date of birth as well as emigration and death (if applicable) were retrieved from the Danish Civil Registration system, which ensures high-fidelity subject identification and matching with respect to emigration and death (12, 16).

### Participants

The study population included subjects alive and residing in Denmark with no emigration history on January 1^st^ 2008. Index date was set as date of diabetes classification for case subjects and a “dummy” date was set for each control subject with respect to emigration and death, i.e. control subjects had to be at risk (alive and Danish resident) at the time of index date to be included. We excluded subjects with classified diabetes before January 1^st^ 2008, those with type 1 diabetes and individuals of age below 50 years at index date (**Supplemental Figure S1**). A 50 years age cut off was chosen as the average age for menopause in Denmark is 51.7 years and the risk of osteoporosis increases afterwards (17). In addition, 12 subjects had misinformed death date and were excluded. Thus, the cohort included adult individuals with age ≥ 50 years without diabetes and new-onset type 2 diabetes between January 1^st^ 2008 and December 31^st^ 2018.

### Identification of Type 2 Diabetes Case Subjects

In order to classify subjects with type 2 diabetes, we identified all subjects with diabetes mellitus between 2008-2018 either by any ICD-10 code (main or secondary) related to diabetes (E10, E11, E12, E13, E14, G63.2, H28.0, H36.0, M14.2, O24, R73) or by an ATC code of glucose-lowering drugs used in diabetes (A10A or A10B) based on a previously published algorithm (18–20). Thereby, all people with diabetes were defined either from a hospital visit or by prescription of glucose-lowering drugs. The diabetes diagnosis and concordance between actual use and prescription of diabetes related medications are in general high (21–26). The diabetes cohort was further classified in type 1 and type 2 diabetes. In Denmark, all patients with type 1 diabetes will eventually be in contact with the hospital and no other glucose-lowering drugs than insulin are recommended. Consequently, type 1 diabetes was defined by at least one E10 ICD-10 code (type 1 diabetes) and at least one A10A ATC code (insulins and analogues) and no A10B ATC code (blood glucose-lowering drugs exclusive insulins); all other individuals with diabetes were classified as type 2 diabetes.

#### Selection of Population-Based Control Subjects

Three control subjects without diabetes mellitus were randomly selected for each case subject and matched by sex and year of birth in order to ensure age and gender adjustment. The control subjects were selected using the incidence-density sampling technique, i.e. control subjects had to be alive and at risk of diabetes at the time the corresponding case was diagnosed with diabetes (time of case occurrence).

### Exposure; Alendronate Use

All prescriptions in Denmark are logged, stored and linked to the unique civil registry number. The prescription database includes data on redeemed drugs and corresponding dates, doses and pack sizes according to the ATC classification system (27). Within the database, we identified all prescriptions of alendronate with the ATC code “M05BA04”. For alendronate exposure, *ever* use (yes/no), *effective* use (cumulative drug dose), and *compliance* were recorded. *Ever* use was defined as any prescription of alendronate before the index date. Effective use was calculated using a Defined Daily Dose (DDD) of 10 mg, based on the World Health Organization Collaborating Centre for Drug Statistics Methodology. To calculate treatment duration, the number of daily doses at the last dispensation date was added to this date, and the date of first drug dispensation was subtracted. Compliance was then assessed using the medication possession ratio (MPR); by dividing the cumulative dose (DDDs) by the treatment duration. MPR was grouped in <0.5, 0.5-0.8 and ≥0.8, the latter being defined as compliant use.

### Identification of Potential Confounding Factors

Potential and measurable risk factors related to type 2 diabetes and alendronate use were selected based on available literature. We identified potential confounders by means of ICD-10 and ATC codes in the period before index date starting from the 1^st^ of January 1998 to index date (**Supplemental Table S2** for specifications).

As a proxy of smoking status, we used ICD-10 codes related to lung diseases, of which some were directly and others indirectly associated with tobacco exposure, as well as nicotine poisoning and psychiatric tobacco-related diagnoses (20). In addition, we identified ATC codes corresponding to treatments for tobacco dependence (ever), e.g. nicotine replacement therapy, or drugs for obstructive airway diseases (after the age of 40). Due to potential underestimation, we classified this factor as *heavy smoking*. We evaluated alcohol consumption by either one relevant ICD-10 or ATC code covering diseases and drugs with direct affiliation to alcohol, e.g. intoxication, alcohol abuse, alcoholic liver disease, alcoholic cardiomyopathy, alcoholic polyneuropathy, alcoholic gastritis, alcohol-induced pancreatitis or alcohol related psychiatric disorders etc. (28). We classified this factor as *alcohol abuse.* Obesity was evaluated by ICD-10 codes of obesity or use of anti-obesity pharmaceuticals by ATC codes. Information on chronic and acute pancreatitis were obtained from ICD-10 codes. Hyper- and hypothyroidism were assessed by either ICD-10 or ATC codes. Comorbidity was assessed by use of Charlson Comorbidity Index (CCI) (29) based on discharge diagnoses registered by ICD-10 codes (**Supplemental Table S3**).

Data on socioeconomic status was obtained from Statistics Denmark. We assessed income as the amount of DKK (Danish kroner) from the year preceding the year of index and adjusted for inflation to a 2018 level using the consumer price index from Statistics Denmark. Lastly, we converted the income to euro € at a rate of 1 € = 7.467 DKK (exchange rate December 2018) and grouped into quintiles for analysis. Marital status was available through the Danish Civil Registration System and assed from the year prior the year of index. It was defined and grouped according to the classification from Statistics Denmark: married, divorced, widowed or unmarried.

### Statistical Analysis

Outcome and exposure were binary variables of type 2 diabetes (case vs. control) and alendronate use, respectively. Exposure was further grouped in categorical variables of duration intervals and compliance. Subject characteristics in tables are presented as numbers and percentages (%), means and standard deviations (SD), % and SD or medians and interquartile range (IQR). In addition, 95% confidence intervals (CI) were calculated, either from means of continues outcomes or proportions of binary outcomes and presented in the text. Unpaired t-test, Chi-square test and Wilcoxon Mann-Whitney median test were performed to compare continuous and dichotomous characteristics between cases and controls. A conditional logistic regression model was used to estimate the effect of alendronate exposure–*ever* use, *effective* use and *compliant* use, respectively–on type 2 diabetes as odds ratios (OR) with 95% CI. A trend test (conditional logic regression model) was performed on effective use, excluding non-users from the analysis to evaluate a possible dose-response relationship between longer duration of alendronate use and risk of type 2 diabetes. We conducted sensitivity analyses excluding heavy smokers, alcohol abusers, prior pancreatitis, glucocorticoid users, obese individuals and those with age above 65. All analyses were conducted in STATA 16.1 (StataCorp, College Station, Texas, US).

## Results

### Study Population Characteristics

**Supplemental Figure S1** presents a flow diagram of the study population selection process. A total of 654,352 individuals were included in the study (163,588 case subjects and 490,764 control subjects). The distribution of sex (55% male subjects) and mean age (66.7 years) were equal among cases and controls confirming a balanced matching. Descriptive subject characteristics can be found in **Table 1**. Subjects with type 2 diabetes were more likely to be heavy smokers, alcohol abusers and obese compared to controls. In addition, pancreatitis, hyperthyroidism, hypothyroidism and previous use of glucocorticoids were more prevalent among subjects with type 2 diabetes compared to control subjects. Lastly, people with type 2 diabetes had a higher degree of comorbidity compared to controls.

**Table 1:** Characteristics of case subjects (type 2 diabetes) and control subjects.

	All subjects	Type 2 diabetes	Control subjects	*P*-value*
	n = 654,352	n = 163,588	n = 490,764	
**Age (years)**, mean ± SD	66.67 ± 10.00	66.67 ± 10.00	66.67 ± 10.00	–
**Age category (years)**, n (%)				–
50-59	198,452 (30.33)	49,613 (30.33)	148,839 (30.38)	–
60-69	231,028 (35.31)	57,757 (35.31)	173,271 (35.31)	–
70-79	161,268 (24.65)	40,317 (24.65)	120,951 (24.65)	–
≥ 80	63,604 (9.72)	15.901 (9.72)	47,703 (9.72)	–
**Sex**, % ± SD				–
Female	44.89 ± 0.50	44.89 ± 0.50	44.89 ± 0.50	
Male	55.11 ± 0.50	55.11 ± 0.50	55.11 ± 0.50	
**Heavy Smoking**, % ± SD	25.84 ± 0.44	32.69 ± 0.47	23.56 ± 0.42	< 0.01
**Alcohol abuse**, % ± SD	4.50 ± 0.21	6.40 ± 0.24	3.87 ± 0.19	< 0.01
**Obesity**, % ± SD	8.80 ± 0.28	17.14 ± 0.38	6.03 ± 0.24	< 0.01
**Pancreatitis**, % ± SD	0.67 ± 0.08	1.61 ± 0.13	0.36 ± 0.06	< 0.01
**Hyperthyroidism**, % ± SD	2.35 ± 0.15	2.96 ± 0.17	2.15 ± 0.14	< 0.01
**Hypothyroidism**, % ± SD	4.85 ± 0.21	6.0 ± 0.24	4.45 ± 0.21	< 0.01
**Glucocorticoid use**, % ± SD	26.88 ± 0.44	31.99 ± 0.47	25.17 ± 0.43	< 0.01
**Hypertension**	57.68 ± 0.49	76.43 ± 0.42	51.42 ± 0.50	< 0.01
**CCI**, mean ± SD	0.51 ± 1.18	0.88 ± 1.53	0.38 ± 1.00	< 0.01
**CCI categories**, n (%)				
0-0.99	490,586 (74.97)	96,372 (58.91)	395,214 (80.33)	< 0.01
1-1.99	75,546 (11.55)	30,758 (18.80)	44,788 (9.13)	< 0.01
≥ 2	88,220 (13.48)	36,458 (22.29)	51,762 (10.55)	< 0.01
**Income**, € in thousands, median (IQR)	30,9 (22,1-47,9)	28,6 (21,4-42,8)	32,1 (22,4-49,5)	<0.01
**Income**, € in thousands				
1^st^ Quintile, median (IQR)	16,4 (14,1-18,3)	16,3 (14,0-18,2)	16,4 (14,2-18,3)	<0.01
2^nd^ Quintile, median (IQR)	23,9 (22,2-25,4)	24,0 (22,2-25,5)	23,9 (22,1-25,4)	<0.01
3^rd^ Quintile, median (IQR)	30,9 (28,7-33,9)	31,0 (28,6-33,6)	31,0 (28,8-34,0)	<0.01
4^th^ Quintile, median (IQR)	43,9 (40,4-47,9)	44,0 (40,2-48,0)	44,0 (40,4-47,9)	<0.01
5^th^ Qiuntile, median (IQR)	66,2 (58,2-83,1)	64,8 (57,6-80,3)	66,4 (58,3-83,8)	<0.01
**Marital status**, n (%)				
Married	400,477 (61.20)	93,176 (57.96)	307,301 (62.62)	<0.01
Divorced	65,984 (10.08)	17,781 (10.87)	48,203 (9.82)	<0.01
Unmarried	91,784 (14.03)	25,687 (15.70)	66,097 (13.47)	<0.01
Widowed	93,172 (14.24)	25,209 (15.41)	67,963 (13.85)	<0.01
Unknown	2,935 (0.45)	1,735 (1.06)	1,200 (0.24)	<0.01

All characteristics were evaluated in the time from 1998 until index date. Data are presented as numbers (n, %), mean with SD or median with IQR. *P-values represent analyses by Chi2-test or Wilcoxon Mann-Whitney median test, significance level was set at 5%.

Regarding socioeconomic status, 2,935 subjects had unknown information on social status; of these, 59.11% were subjects with type 2 diabetes, and 40,89% were control subjects. Control subjects were more likely to be married than subjects with type 2 diabetes (62.62% [95% CI 62.48-62.75] vs 56.96% [95% CI 56.72-57.20]. In addition, control subjects had a higher income before index date compared to people with type 2 diabetes. Lastly, a higher proportion of control subjects were in the 5^th^ income quintile compared to type 2 diabetes [21.72% (95% CI 21.60-21.83) vs 14.85% (96% CI 14.68-15.03)].

### Characteristics of Alendronate Users

Descriptive characteristics of people exposed vs unexposed to alendronate can be found in **Supplemental Table S4**. In total, we identified 31,976 users of alendronate prior to or at index date with a median exposure time of 2.55 years (IQR 0.75-5.26). Of these, 25,169 were control subjects and 6,807 were type 2 diabetes patients corresponding to 5.13% (95% CI 5.07-5.19) and 4.16% (95% CI 4.06-4.26), respectively. Median exposure time was 2.31 years (IQR 0.68-4.98) for type 2 diabetes patients and 2.61 years (IQR 0.78-5.32) for control subjects. In total, 20,786 subjects (65.01%) were still users of alendronate at index date with a higher proportion among control subjects; corresponding to 66.32% (95% CI 65.73-66.90) of control subjects and 60.14% (95% CI 58.97-61.31) of type 2 diabetes subjects.

The proportion of females with alendronate use was in general higher than males with alendronate use [8.95% (95% CI 8.84-9.05) vs 1.58% (95% CI 1.54-1.62)]. However, the proportion of alendronate users was lower among female type 2 diabetes subjects than among female control subjects [78.60% (95% CI 77.60-79.56) vs 83.15% (95% CI 82.68-83.61)]. The highest percentage of alendronate users was found in the age group 70-79 years (39.25%) in both patients with type 2 diabetes (38.64%) and control subjects (39.42%). However, male users of alendronate were younger than female users [mean age in years: 67.11 (95% CI 66.86-67.35) vs 70.21 (95% CI 70.09-70.32)].

#### Ever Use of Alendronate

The ORs for developing incident type 2 diabetes after alendronate use are presented in **Table 2**. Patients with type 2 diabetes were less likely than matched control subjects to have ever used alendronate and the association became more pronounced after multiple adjustment. The crude OR was significantly lower among those who used alendronate at index date compared to those who had stopped prior to index date [OR: 0.82 (95% CI 0.73-0.93)] but became insignificant after adjustment (OR: 0.94 [95% CI 0.81-1.09]).

**Table 2:** Risk of type 2 diabetes presented as crude and adjusted ORs grouped in ever, effective and compliant users.

	Cases, n (%)	Controls, n (%)	Crude OR (95% CI)	Adjusted OR (95% CI)
	n = 163,588 (100)	n = 490,764 (100)	(age/sex-match)	All confounders¥
Never users of alendronate	156,781 (95.84)	465,595 (94.87)	1.00 (ref.)	1.00 (ref.)
**Ever users of alendronate**	6,807 (4.16)	25,169 (5.13)	**0.79** (0.77-0.81)	**0.64** (0.62-0.66)
**Effective use**				
< 6 months	1,657 (1.01)	5,563 (1.09)	1.00 (ref.)	1.00 (ref.)
0.5-1.9 years	1,945 (1.19)	6,751 (1.38)	0.93 (0.87-1.01)	0.94 (0.87-1.02)
2-3.9 years	1,422 (0.87)	5,605 (1.14)	**0.82** (0.76-0.89)	**0.87** (0.80-0.95)
4-5.9 years	827 (0.51)	3,656 (0.74)	**0.73** (0.66-0.80)	**0.79** (0.72-0.88)
6-7.9 years	516 (0.32)	2,003 (0.41)	**0.83** (0.74-0.93)	0.89 (0.79-1.00)
>8 years	440 (0.27)	1,791 (0.36)	**0.78** (0.70-0.88)	**0.84** (0.74-0.95)
**Compliant use**				
MPR < 0.5	564 (8.29)	1,750 (6.95)	1.00 (ref.)	1.00 (ref.)
MPR 0.5-0.8	1,090 (16.01)	3,835 (15.24)	**0.88** (0.79-0.99)	0.93 (0.82-1.05)
MPR > 0.8	5,153 (75.70)	19,584 (77.81)	**0.82** (0.74-0.90)	0.90 (0.80-1.00)

Conditional logistic regression analysis of ORs (95% CI) for development of type 2 diabetes when exposed to alendronate.

¥Adjusted for smoking, alcohol, obesity, pancreatitis, hypothyroidism, hyperthyroidism, use of glucocorticoids, CCI, income and marital status. Estimates in bold represent p < 0.05.

Stratification by sex and the ORs of incident type 2 diabetes are presented in **Table 3**. Female subjects were more likely to ever use alendronate compared to male subjects, and the odds for type 2 diabetes were correspondingly lower among female ever users (**Table 3**).

**Table 3:** Risk of type 2 diabetes stratified by sex.

	Females		Males	
	Crude OR	Adjusted OR	Crude OR	Adjusted OR
Never users	1.00 (ref.)	1.00 (ref.)	1.00 (ref.)	1.00 (ref)
**Ever users**	**0.74** (0.72-0.76)	**0.60** (0.58-0.62)	1.03 (0.97-1.10)	**0.71** (0.67-0.77)
**Effective use**				
< 6 months	1.00 (ref.)	1.00 (ref.)	1.00 (ref.)	1.00 (ref.)
0.5-1.9 years	0.95 (0.87-1.03)	0.96 (0.87-1.05)	0.90 (0.77-1.05)	0.90 (0.77-1.06)
2-3.9 years	**0.82** (0.75-0.90)	**0.88** (0.87-0.97)	0.87 (0.73-1.03)	0.87 (0.73-1.05)
4-5.9 years	**0.75** (0.68-0.84)	**0.82** (0.74-0.92)	**0.70** (0.56-0.87)	**0.72** (0.57-0.90)
6-7.9 years	**0.84** (0.74-0.95)	0.89 (0.78-1.01)	0.91 (0.69-1.19)	0.96 (0.73-1.28)
>8 years	**0.83** (0.73-0.94)	0.88 (0.77-1.01)	**0.70** (0.51-0.98)	0.73 (0.51-1.03)
**Compliant use**				
MPR < 0.5	1.00 (ref.)	1.00 (ref.)	1.00 (ref.)	1.00 (ref.)
MPR 0.5-0.8	**0.85** (0.74-0.96)	0.91 (0.79-1.04)	**0.88** (0.79-0.99)	0.93 (0.82-1.05)
MPR > 0.8	**0.80** (0.71-0.89)	0.90 (0.79-1.01)	**0.82** (0.74-0.90)	0.90 (0.80-1.00)

Conditional logistic regression analysis of OR (95% CI) for development of type 2 diabetes when exposed to alendronate.

¥Adjusted for smoking, alcohol, obesity, pancreatitis, hypo- and hyperthyroidism, use of glucocorticoids, CCI, income and marital status. Estimates in bold represent p < 0.05.

In an analysis including all used types of oral administrated bisphosphonates, i.e. etidronate (ATC-code M05BA01), risedronate (ATC-code M05BA07), ibandronate (ATC-code M05BA06) and pamidronate (ATC-code M05BA03), the results did not change [crude OR: 0.79 (95% CI 0.77-0.81), adjusted OR: 0.63 (95% CI 0.61-0.65)].

#### Alendronate Duration and Compliance

**Figure 1:**
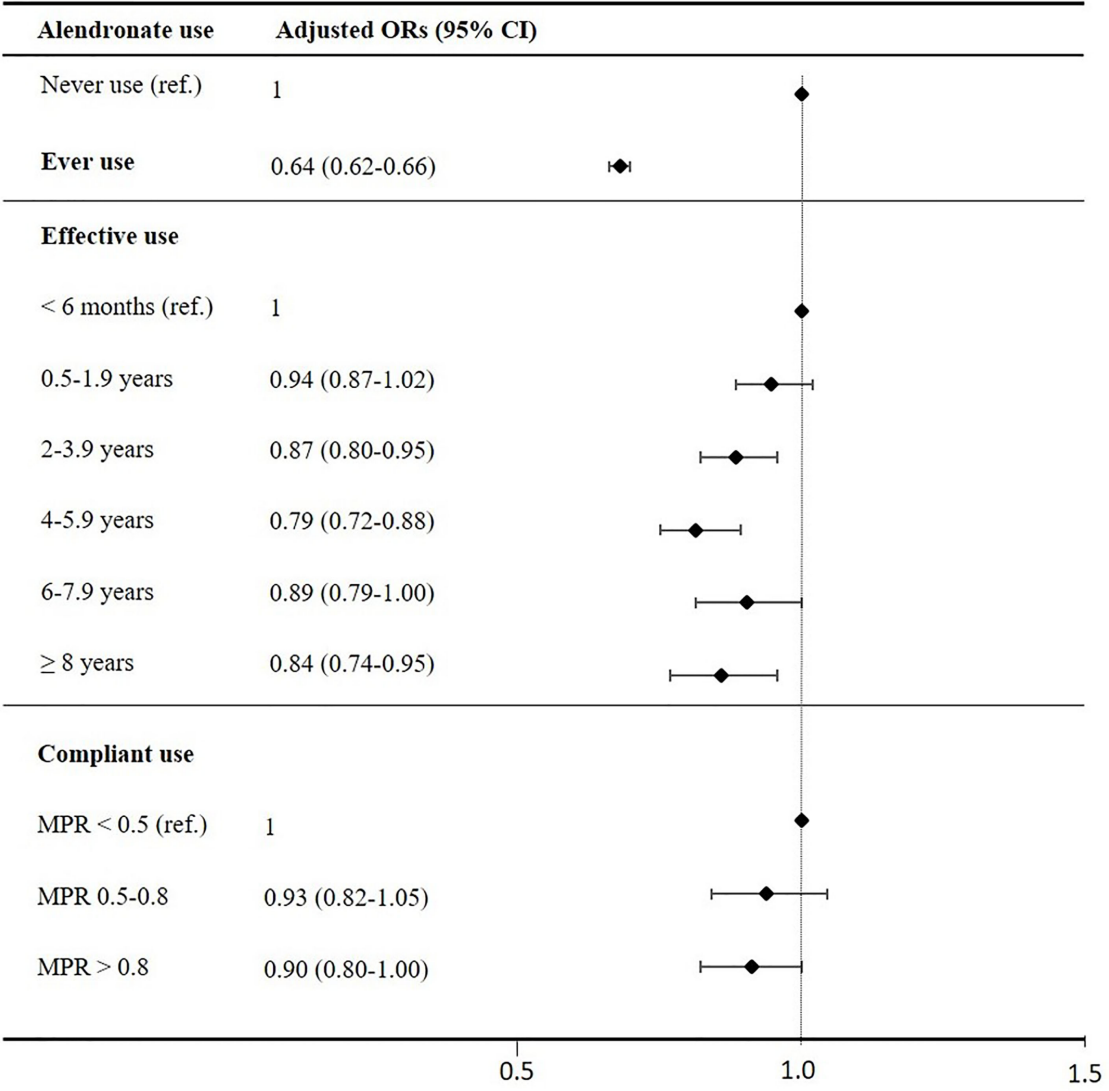
Adjusted ORs for development of type 2 diabetes. ORs are adjusted for heavy smoking, alcohol abuse, obesity, pancreatitis, hyperthyroidism, hypothyroidism, steroid use, CCI, income, social status and presented with lower and upper CI 95% as error bars. The lowest categories (never users, effective users with use below 6months and compliant users with MPR below 0.5) as reference line (OR=1).

**Table 2** presents crude and adjusted ORs for *effective* use (<6 months as reference) and *compliant* use (MPR<0.5 as reference), which are also illustrated in **Figure 1**. The ORs for incident type 2 diabetes decreased with longer effective use and the lowest OR was found among effective alendronate use of 4-6 years compared to those with less than 6 months of use. The trend test revealed a dose-response relationship between longer effective use in years and lower risk of type 2 diabetes (p<0.007). Additionally, a trend towards a more pronounced association with compliant users were observed (MPR>0.8, p=0.052). The risk of type 2 diabetes was lower among those who used alendronate <6 months compared to never users of alendronate [adjusted OR: 0.70 [95% CI 0.66-0.74)].

In female subjects, ever use of alendronate was significantly associated with a decreased OR after 2-6 years of use (**Table 3**). In male subjects, use of alendronate was associated with a decreased OR after 4-6 years. In addition, the crude ORs were significantly lower in compliant female alendronate users compared to non-compliant users, however, the ORs became insignificant after multiple adjustment.

#### Sensitivity Analyses

In sensitivity analyses with the exclusion of obese subjects, alcohol users, pancreatitis, steroid users or heavy smokers ever use of alendronate still revealed a lower OR of incident type 2 diabetes. In particular, the OR decreased further when excluding heavy smokers (adjusted OR: 0.59; 95% CI 0.57-0.62). Similarly, when excluding all individuals aged above 65 years (n=339,604), the OR remained significantly low [adjusted OR: 0.56 (95% CI 0.52-0.61)]. Stratification by hypertension status revealed a lower OR among people without hypertension [adjusted OR: 0.53 (95% CI 0.47-0.59)]. No change in OR was found between people with and without dyslipidemia.

## Discussion

In this large nationwide nested case-control study, we found that patients with type 2 diabetes were less likely than matched control subjects to have ever used alendronate. The largest risk reduction observed was almost 40% among ever users of alendronate compared to non-users. In addition, we present a significant and prominent dose-dependent association between longer effective use and decreased risk of type 2 diabetes. To our knowledge, this is the first case-control study investigating the association between alendronate use and type 2 diabetes.

The risk of incident type 2 diabetes was significantly lower among those with less than 6 months of alendronate use compared to never users. This may suggest that alendronate acts somewhat promptly on glucose metabolism, with its effects becoming more prominent after several years. We chose to only include alendronate as exposure, as this is first line recommendation and the most frequent used bisphosphonate in both research and clinics settings. Few clinical trials have investigated the association between alendronate use and glucose metabolism. Fard et al. conducted a randomized controlled trial with 60 postmenopausal women aged 45-60 years enrolled to receive either 70 mg alendronate per week or placebo for 12 weeks (5). They found reduced fasting plasma glucose, insulin concentration and increase in insulin sensitivity measured by the Matsuda Index in the alendronate group (both compared to baseline and to the control group). This short 12-week intervention period confirms our finding of a possible protective effect already after 6 months. How alendronate influences on glucose metabolism is not clarified. *In vitro* studies suggest that alendronate decreases adipogenesis and activates lipolysis (30, 31), conditions that may be altered in subjects with decreased insulin sensitivity. Furthermore, a bone-resorption-specific impact on insulin signaling by Osteocalcin has been suggested, though the evidence for an effect in humans is very limited and the hypothesis is based on an animal model (32). Schwartz et al. performed *post hoc* analyses of three randomized controlled trials with 3-4 years of follow-up but did not find any changes in diabetes incidence after treatment with alendronate, zoledronic acid or denosumab (33). However, the daily administration of alendronate was only 5 mg during the first 2 years and increased to 10 mg for the last 2 years. According to our dose-dependent findings 5 mg daily may be an inadequate dose to reveal a significant effect of alendronate on fasting blood glucose and risk of type 2 diabetes.

In Denmark, it is recommended to re-evaluate treatment after 5 years to consider discontinuing based on BMD evaluation. Individuals using alendronate for a longer time were in general more comorbid estimated by a CCI. It is possible that individuals with osteoporosis but without pre-diabetes have a higher possibility of discontinuing treatment after 5 years compared to those with greater risk of type 2 diabetes as a result of potentially healthier bones. This may explain why the OR increased in the 6-8 years exposure group. Unfortunately, we did not have access to neither blood samples nor bone scans and consequently no measures of hemoglobin A1c or BMD were available. However, we found that the risk of developing type 2 diabetes were lower among those who continued alendronate treatment, suggesting a possible sustained protection together with long term effects, corresponding to a known long half-life of alendronate (34).

Although research concerning alendronate and type 2 diabetes are conflicting, our results are consistent with several previous studies. Vestergaard et al. conducted a cohort study on 103,562 individuals exposed to alendronate and found an OR of 0.69 (95% CI 0.57-0.83) for type 2 diabetes after adjustment for corticosteroid use (6). In addition, the study reported a decreasing risk of developing type 2 diabetes with increasing doses of alendronate. Another cohort study by Toulis et al. suggested a 50% risk reduction of type 2 diabetes among users of alendronate for more than 1 year compared to those who did not use alendronate (9). We chose to include all with a prescription of alendronate as exposed individuals and found significantly lower adjusted odds for diabetes after 2 years (compared to those with less than 6 months of use). In addition, we were able to adjust for several relevant confounders, including heavy smoking, alcohol abuse, socioeconomic status and several other comorbidities and medication uses.

One notable strength in the present study is the high quality and validity of the Danish National Registers based on the unique identification number assigned to all Danish citizens (12, 14, 35, 36). Furthermore, the identification of people diagnosed with diabetes in Denmark was nationwide without any selection bias. We present data from a large cohort that enabled us to match exactly 3 controls to every case randomly by incidence-density sampling on age and gender to eliminate bias and ensure uniform risk and exposure time. We expected subjects exposed to alendronate to be relatively unhealthy compared to those who did not receive alendronate due, for instance, to risk factors for osteoporosis (37), e.g. smoking as presented in **Supplemental Table S4**. Contrarily, it is possible that people with osteoporosis and relatively longer exposure duration are healthier, have higher tolerance for alendronate, and may have lower BMI (38) and, consequently, lower risk of type 2 diabetes. This may give rise to healthy survivor bias as seen in many previous cohort studies, and so we chose a case-control setup to minimize that bias. Our sensitivity analyses suggest that the risk of type 2 diabetes decrease further when heavy smokers and people with hypertension are excluded from the analysis. Smoking is a risk factor for type 2 diabetes but as are obesity, alcohol and pancreatitis of which the ORs did not change after sensitivity analyses. It may be that smoking impacts on alendronates mechanism of action, as has been addressed recently (39), although this warrants further research.

One important limitation of this case-control study is the retrospective design which hinders the inference of causation between alendronate exposure and type 2 diabetes. Another limitation is the data collection and diabetes classification. We excluded people from the type 2 diabetes group if they had ever received an E10 (Type 1 diabetes mellitus) diagnosis and no glucose-lowering drugs other than insulins (ATC A10B). Thus, people initially misdiagnosed as having type 1 diabetes or possibly severe cases of type 2 diabetes were lost in this investigation. All Danish citizens with type 1 diabetes will eventually be in contact with the hospital and will thereby be given an ICD-10 E10 code. In contrast, general practitioners outside the hospital will most often be responsible for treatment of people with type 2 diabetes. Thus, only complicated cases of type 2 diabetes will be treated in the hospital and receive an ICD-10 E11 (type 2 diabetes mellitus) code. However, individuals who have never been in contact with the hospital but have ever received A10B medications were classified as type 2 diabetes and included in the cohort. By this classification, we were unable to identify naïve or mild cases of type 2 diabetes, e.g. people who have never received an ICD code or glucose-lowering drugs and have thus been treated with life-style interventions only. Although the Danish registries contain a wide range of information, we did not have access to over-the-counter-medicine, e.g. vitamin D supplementation, and we were unable to correct for lifestyle factors such as diet and exercise, which might have been associated with our identified outcome and exposure. In addition, the registries did not include data on smoking habits and alcohol consumptions; however, we estimated some of these baseline characteristics by ICD-10 and ATC codes. Consequently, we only obtained these characteristics from those with already developed concomitant disease or with prescribed medical therapy. We have information on diagnoses of adiposity, but not information on BMI in the included people. The cohort consists of divergent patient groups and people with osteoporosis may have a lower BMI with possible protection against development of type 2 diabetes (40), a bias that may lead to confounding by indication.

This study supports the hypothesis of an interaction between glucose homeostasis and bone metabolism. It is possible that people with osteoporosis and increased risk of type 2 diabetes could benefit from alendronate concerning the risk of developing type 2 diabetes after osteoporosis diagnosis. In addition, it is still unclear whether other anti-resorptive therapies, e.g., denosumab, show similar tendencies.

In conclusion, our data support previous studies suggesting a possible protective effect of alendronate on development of type 2 diabetes in a dose-dependent manner. In addition, it seems that smoking may suppress this protective effect. However, the underlying mechanism needs further exploration, and so we propose future research to prospectively evaluate glucose metabolism in people with and without type 2 diabetes exposed to alendronate.

## Data Availability Statement

The data analyzed in this study is subject to the following licenses/restrictions: All authorized Danish research organizations can apply for access to the registries. An ethics committee approval is not required for epidemiological studies in Denmark, as we had no access to personally identifiable information. However, the registries are subject to control by the Danish Data Protection Agency. Requests to access these datasets should be directed to https://www.dst.dk/en.

## Author Contributions

All authors contributed to the article according to the ICJME requirements for co-authorship. All authors had full access to all data used in the study, critically revised the paper for intellectual content and approved submitted versions and the final version of the paper. RV and PV designed the study. RV performed data management and statistical analyses with assistance from PV. RV and PV interpreted the data. RV wrote the paper. ZA-M and JS-L made critical revisions of data management, design, data interpretation and reviewed the manuscript.

## Funding

This work was supported by Steno Collaborative grant, Novo Nordisk Foundation, Denmark (Grant no. NNF18OC0052064).

## Conflict of Interest

The authors declare that the research was conducted in the absence of any commercial or financial relationships that could be construed as a potential conflict of interest.

## Publisher’s Note

All claims expressed in this article are solely those of the authors and do not necessarily represent those of their affiliated organizations, or those of the publisher, the editors and the reviewers. Any product that may be evaluated in this article, or claim that may be made by its manufacturer, is not guaranteed or endorsed by the publisher.
