# Supplementary material for: Alendronate Use and Risk of Type 2 Diabetes: A Nationwide Danish Nested Case-Control Study
Source: Front Endocrinol (Lausanne). 2021 Nov 19;12:771426. doi: 10.3389/fendo.2021.771426 (PMC8640922; doi:10.3389/fendo.2021.771426)

Supplemental Table S1, STROBE Checklist for observational studies.

| **Section/Topic** | Item No | Recommendation | Reported on Page No |
| --- | --- | --- | --- |
| **Title and abstract** | 1 | (*a*) Indicate the study’s design with a commonly used term in the title or the abstract | 1 |
|  |  | (*b*) Provide in the abstract an informative and balanced summary of what was done and what was found | 2 |
| Introduction | | | |
| Background/rationale | 2 | Explain the scientific background and rationale for the investigation being reported | 3 |
| Objectives | 3 | State specific objectives, including any prespecified hypotheses | 3 |
| Methods | | | |
| Study design | 4 | Present key elements of study design early in the paper | 3 |
| Setting | 5 | Describe the setting, locations, and relevant dates, including periods of recruitment, exposure, follow-up, and data collection | 3-4 |
| Participants | 6 | (*a*) *Cohort study*—Give the eligibility criteria, and the sources and methods of selection of participants. Describe methods of follow-up  *Case-control study*—Give the eligibility criteria, and the sources and methods of case ascertainment and control selection. Give the rationale for the choice of cases and controls  *Cross-sectional study*—Give the eligibility criteria, and the sources and methods of selection of participants |  |
|  |  | (*b*) *Cohort study*—For matched studies, give matching criteria and number of exposed and unexposed  *Case-control study*—For matched studies, give matching criteria and the number of controls per case | 5 |
| Variables | 7 | Clearly define all outcomes, exposures, predictors, potential confounders, and effect modifiers. Give diagnostic criteria, if applicable | 6 |
| Data sources/measurement | 8* | For each variable of interest, give sources of data and details of methods of assessment (measurement). Describe comparability of assessment methods if there is more than one group | 7 |
| Bias | 9 | Describe any efforts to address potential sources of bias |  |
| Study size | 10 | Explain how the study size was arrived at | 6 |
| Quantitative variables | 11 | Explain how quantitative variables were handled in the analyses. If applicable, describe which groupings were chosen and why | 5, 8 |
| Statistical methods | 12 | (*a*) Describe all statistical methods, including those used to control for confounding | 8 |
|  |  | (*b*) Describe any methods used to examine subgroups and interactions | 8 |
|  |  | (*c*) Explain how missing data were addressed | - |
|  |  | (*d*) *Cohort study*—If applicable, explain how loss to follow-up was addressed  *Case-control study*—If applicable, explain how matching of cases and controls was addressed  *Cross-sectional study*—If applicable, describe analytical methods taking account of sampling strategy | 5 |
|  |  | (*e*) Describe any sensitivity analyses | 8 |

| **Section/Topic** | Item No | Recommendation | Reported on Page No |
| --- | --- | --- | --- |
| Results | | | |
| Participants | 13* | (a) Report numbers of individuals at each stage of study—eg numbers potentially eligible, examined for eligibility, confirmed eligible, included in the study, completing follow-up, and analysed | 9 |
|  |  | (b) Give reasons for non-participation at each stage |  |
|  |  | (c) Consider use of a flow diagram | S1 |
| Descriptive data | 14* | (a) Give characteristics of study participants (eg demographic, clinical, social) and information on exposures and potential confounders | 9 |
|  |  | (b) Indicate number of participants with missing data for each variable of interest | - |
|  |  | (c) *Cohort study*—Summarise follow-up time (eg, average and total amount) | - |
| Outcome data | 15* | *Cohort study*—Report numbers of outcome events or summary measures over time |  |
|  |  | *Case-control study—*Report numbers in each exposure category, or summary measures of exposure | 10 |
|  |  | *Cross-sectional study—*Report numbers of outcome events or summary measures |  |
| Main results | 16 | (*a*) Give unadjusted estimates and, if applicable, confounder-adjusted estimates and their precision (eg, 95% confidence interval). Make clear which confounders were adjusted for and why they were included | 11,12 |
|  |  | (*b*) Report category boundaries when continuous variables were categorized | 11 |
|  |  | (*c*) If relevant, consider translating estimates of relative risk into absolute risk for a meaningful time period |  |
| Other analyses | 17 | Report other analyses done—eg analyses of subgroups and interactions, and sensitivity analyses | 12 |
| Discussion | | | |
| Key results | 18 | Summarise key results with reference to study objectives | 12 |
| Limitations | 19 | Discuss limitations of the study, taking into account sources of potential bias or imprecision. Discuss both direction and magnitude of any potential bias | 15 |
| Interpretation | 20 | Give a cautious overall interpretation of results considering objectives, limitations, multiplicity of analyses, results from similar studies, and other relevant evidence | 14,15 |
| Generalisability | 21 | Discuss the generalisability (external validity) of the study results | 15,16 |
| Other Information | | | |
| Funding | 22 | Give the source of funding and the role of the funders for the present study and, if applicable, for the original study on which the present article is based | 17 |

Supplemental Table S2. Identification of diabetes and confounders.

|  | **ICD-10 codes** | **ATC codes** |
| --- | --- | --- |
| Diabetes mellitus | E10, E11, E12, E13, E14, G63.2, H28.0, H36.0, M14.2, O24, R73 | A10A, A10B |
| Heavy smoking | J41-J44, J47,  Z720, F17, T652 | N07BA, N06AX12.  After age 40: R03A, R03B, R03C, R03DA, R03DB, R03DC, R03DX07 |
| Alcohol abuse | T51, G312, G621, I426, K292, K70, K852, K860, F10 | N07BB01, N07BB03, N07BB05 |
| Obesity | E66 | A08A |
| Hyperthyroidism | E05 | H03B |
| Hypothyroidism | E03 | H03A |
| Glucocorticoid use |  | H02AB |
| Pancreatitis | K86.0, K86.1, K85.0, K85.1, K85.2, K85.3, K85.8, K85.9 |  |
| Hypertension | I10-I13, I15, R03 | C02DB, C02CA, C02AB, C02AC, C03, C08, C07A, C09 |
| Dyslipidemia | E75, E78 | C10 |

Supplemental Table S3. Charlson Comorbidity Index; scoring and ICD codes

| **No., Comorbidity** | **Score** | **ICD-10 codes** |
| --- | --- | --- |
| 1, Acute myocardial infarction | 1 | DI21, DI23, I24.1, I24.8, I24.9 |
| 2, Cardiac insufficiency | 1 | I50, I11.0, I13.0, I13.2 |
| 3, Cardiovascular disease | 1 | I70, I71, I72, I73, I74, I77 |
| 4, Cerebrovascular disease | 1 | I60-I69, G45, G46 |
| 5, Dementia | 1 | F00-F03, F05.1, G30 |
| 6, Chronic pulmonary disease | 1 | J40-J47, J60-J67, J68.4, J70.1, J70.3, J70.4, J82, J84, J85.0, J92, J95.3, J96.1, J98.2, J98.3 |
| 7, Connective tissue disease | 1 | M05-M09, M30-M36, D86 |
| 8, Peptic ulcer | 1 | K22.1, K25-K28 |
| 9, Mild liver disease | 1 | B18, K70.0-K70.3, K709, K71, K73, K74, K75.2-K75.4, B15.9, B16.9, K75.8-K75.9, K76.0 |
| 10, Diabetes mellitus | - | - |
| 11, Hemiplegia | 1 | G81, G82 |
| 12, Nephrological disease | 2 | I12, I13, N02-N04, N07, N11, N12, N14, N18-N19, Q60-Q61 |
| 13, Late-diabetic complications | - | - |
| 14, Solid cancers | 2 | C00-C75 |
| 15, Leukemia | 2 | DC91-DC95 |
| 16, Lymphoma | 2 | C81-C85, C88, C90, C96 |
| 17, Moderate to severe liver disease | 3 | B15.0, B16.0, B16.2, B19.0, K70.4, K72, K76.6, K76.7, I85 |
| 18, Metastatic cancer | 6 | C77-C80 |
| 19, AIDS | 6 | B20-B24 |

Supplemental Table S4, Subjects characteristics of alendronate users and non-users.

|  | **Alendronate ever users**  n = 31,976 | **Alendronate never users**  n = 622,376 | *P*-value* |
| --- | --- | --- | --- |
|  |  |  |  |
| **Age (years),** mean ± SD | 74.52 ± 9.30 | 66.27 ± 9.87 | < 0.001 |
| **Age category (years)**, n (%)  50-59  60-69  70-79  ≥ 80 | 2,367 (7.40)  8,302 (25.96)  12,551 (39.25)  8,756 (27.38) | 196,085 (31.51)  222,726 (35.79)  148,717 (23.90)  54,848 (8.81) | < 0.001 |
| **Sex,** % ± SD  Female  Male | 82.18 ± 0.38  17.82 ± 0.38 | 42.97 ± 0.50  57.03 ± 0.50 | < 0.001  < 0.001 |
| **Type 2 diabetes,** % ± SD | 21.28 ± 0.41 | 25.19 ± 0.43 | < 0.001 |
| **Heavy Smoking,** % ± SD | 40.28 ± 0.49 | 25.10 ± 0.43 | < 0.001 |
| **Alcohol abuse,** % ± SD | 4.35 ± 0.20 | 4.50 ± 0.21 | 0.18 |
| **Obesity,** % ± SD | 7.75 ± 0.27 | 8.86 ± 0.28 | < 0.001 |
| **Pancreatitis,** % ± SD | 1.04 ± 0.10 | 0.65 ± 0.08 | < 0.001 |
| **Hyperthyroidism,** % ± SD | 6.24 ± 0.24 | 2.15 ± 0.14 | < 0.001 |
| **Hypothyroidism,** % ± SD | 9.34 ± 0.29 | 4.62 ± 0.21 | < 0.001 |
| **Glucocorticoid use,** % ± SD | 50.66 ± 0.50 | 25.66 ± 0.44 | < 0.001 |
| **CCI,** mean ± SD | 1.03 ± 1.57 | 0.49 ± 1.15 | < 0.001 |
| **CCI categories,** n (%)  0-1  1-2  >2 | 12,846 (53.78)  4,532 (18.97)  6,509 (27.25) | 477,740 (75.78)  71,014 (11.26)  81,711 (12.96) | < 0.001  < 0.001  < 0.001 |
| **Income,** € in thousands, median (IQR) | 25,9 (19,5-33,3) | 31,3 (22,3-48,4) | <0.001 |
| **Income,** € in thousands, median (IQR)  1^st^ Quintile, median (IQR)  2^nd^ Quintile, median (IQR)  3^rd^ Quintile, median (IQR)  4^th^ Quintile, median (IQR)  5^th^ Qiuntile, median (IQR) | 16,5 (14,7-18,3)  24,0 (22,2-25,6)  30,4 (28,4-33,2)  42,4 (40,4-47,9)  63,4 (57,2-76,0) | 16,3 (14,1-18,3)  23,9 (22,2-25,4)  30,9 (28,7-33,9)  43,9 (40,4-47,9)  66,2 (58,2-83,2) | <0.001  0.004  <0.001  <0.001  <0.001 |
| **Marital status,** n (%)  Married  Divorced  Unmarried  Widowed  Unknown | 11,514 (48.20)  1,330 (5.57)  3.131 (13.11)  7,911 (33.12)  1 (0.00) | 388,963 (61.69)  64,654 (10.25)  88,653 (14.06)  85,261 (13.52)  2,934 (0.47) | <0.001  <0.001  <0.001  <0.001  <0.001 |

Supplemental Figure S1, Flow-diagram of the study group selection process.


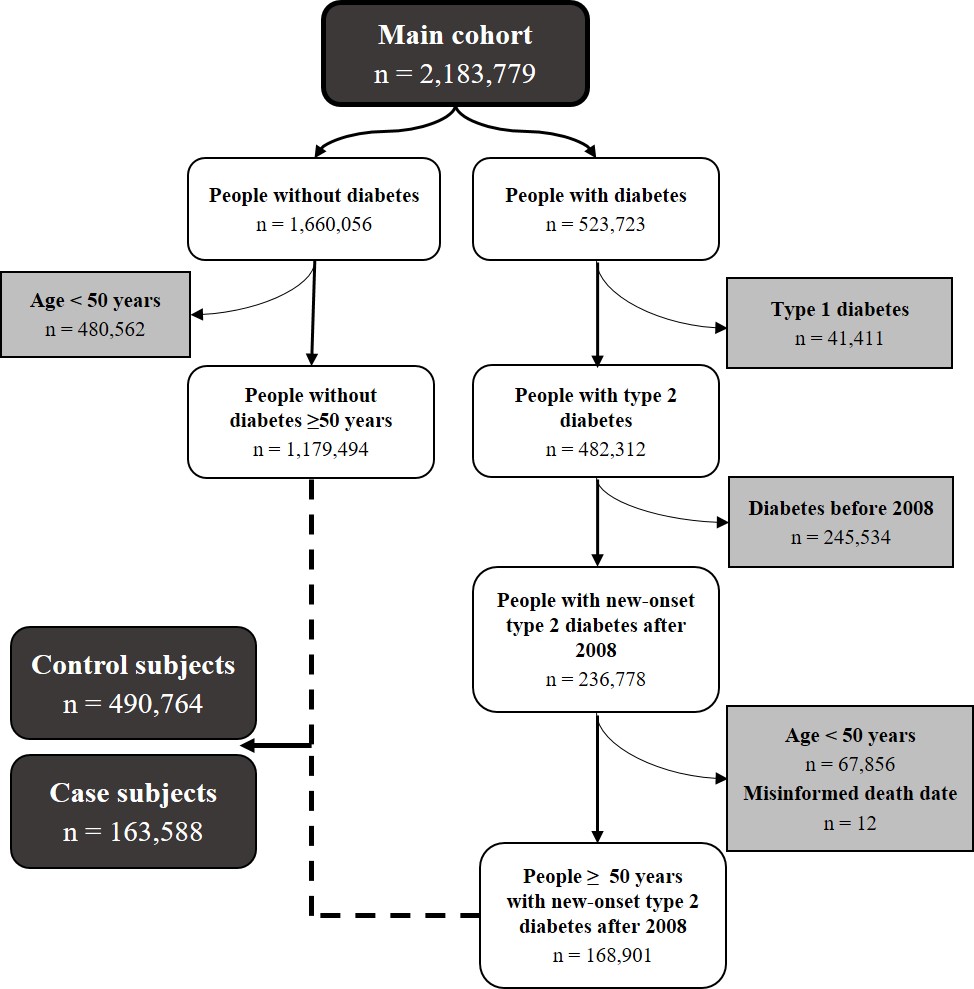

Supplement: Supplementary file 1 [file DataSheet_1.docx]
